# Cross-sectional evaluation of health resource use in patients with functional neurological disorders referred to a tertiary neuroscience centre

**DOI:** 10.1136/bmjno-2023-000606

**Published:** 2024-05-22

**Authors:** Brian William O'Mahony, Robert Nelson-Sice, Glenn Nielsen, Rachael Hunter, Sarah Cope, Niruj Agarwal, Mark J Edwards, Mahinda Yogarajah

**Affiliations:** 1 Institute of Psychiatry at the Maudsley, London, UK; 2 University Hospital Galway, Galway, Ireland; 3 St George's Healthcare NHS Trust, London, UK; 4 Institute of Molecular and Clinical Sciences, St George's University, London, UK; 5 Primary Care and Population Health, University College London, London, UK; 6 South West London and Saint George's Mental Health NHS Trust, London, UK; 7 Department of Neuropsychiatry, South West London and St George's Mental Health NHS Trust, London, UK; 8 St George’s University of London, London, UK; 9 Department of Clinical and Experimental Epilepsy, National Hospital for Neurology and Neurosurgery, London, UK

**Keywords:** functional neurological disorder, conversion disorder, health economics

## Abstract

**Introduction:**

Functional neurological disorder (FND) is a common cause of referral to neurology services. FND has been shown to lead to significant healthcare resource use and is associated with significant disability, comorbidity and distress. This leads to substantial direct, indirect and intangible costs to the patient and society.

**Methods:**

We recruited consecutive patients with FND referred to a tertiary FND specialist clinic. We assessed health and social care resource use in the 6 months preceding their consultation through a modified version of the Client Service Receipt Inventory in the form of a postal questionnaire. The total cost was estimated by combining the number and frequency of health resource use with standard national unit costs. We also assessed indirect costs such as informal care and loss of income.

**Results:**

We collected data on 118 subjects. Patients with comorbid anxiety or depression had higher costs in the preceding 6 months, as did patients who had a longer duration of FND symptoms. Indirect costs were higher than the already substantial direct costs and a large proportion of patients with FND were receiving government support.

**Conclusion:**

This study highlights the high cost of FND to both patients and health systems. Adequate reform of the patient pathway and reorganisation of services to make diagnoses and initiate treatment more quickly would likely reduce these costs.

**WHAT IS ALREADY KNOWN ON THIS TOPIC**

- Patients with a functional neurological disorder (FND) are known to have high healthcare resource utilisation; however, a recent systematic review showed that the literature on healthcare costs is sparse, particularly regarding indirect costs.

**WHAT THIS STUDY ADDS**

- While patients with FND have high direct healthcare costs, their indirect costs appear to be significantly higher. Additionally, those with a longer duration of illness appear to incur higher costs than those with a more recent onset.

**HOW THIS STUDY MIGHT AFFECT RESEARCH, PRACTICE OR POLICY**

- This paper emphasises the importance of service provision and early intervention for patients with FND.

## Introduction

Functional neurological disorder (FND) represents genuine and involuntary neurological symptoms and signs that have characteristic clinical features and represent a problem of voluntary control and perception despite the normal basic structure of the nervous system.^1^ Manifestations of FND are varied, such as decreased or increased movement, loss of sensation, difficulties in speech, abnormal gait or posture, cognitive symptoms and seizure-like episodes (functional seizures (FS)).^1^ FND can have a significant impact on the sufferer’s quality of life.^2^ Patients often present with comorbid psychiatric conditions, with both depression and anxiety occurring in up to 40% of patients with FND.^3 4^

The FND of movement and sensation has a prevalence of roughly 50 per 100 000 population and an incidence of 4–12 per 100 000 population per year. FS contributes a further 1.5–4.9 per 100 000 population per year, with a prevalence of 2–33 per 100 000 population.^5^ Patients with FND make up 9% of neurology admissions^6^ and 16% of neurology clinic referrals.^7^ Delayed diagnoses of FND lead to worse outcomes for patients,^3^ as well as preventable costs, such as missed work, general practitioner (GP) and specialist appointments, and investigations. Diagnostic uncertainty in the midst of ongoing symptoms can lead to intangible costs, such as decreased quality of life (QOL). These costs carry a burden on patients, clinicians, healthcare systems and the economy.

The costs of FND (and other medical conditions) can be separated into direct and indirect costs. Direct costs represent resources used for healthcare (eg, the cost of investigations and time spent on assessments by a doctor), as well as out-of-pocket costs to the patient. Indirect costs represent productivity losses arising from morbidity-related sickness absence (eg, loss of employment and cost of childcare while hospitalised). Direct and indirect costs together constitute the economic burden of FND, which can be estimated by measuring the monetary valuation of healthcare utilisation and lost productivity in patient samples.

The literature concerning the economic cost of FND is sparse, and any conclusions that may be drawn from it are limited by the heterogeneity of the studies that focus on the topic. Studies vary in the costs included in their analysis, with many focusing only on hospital costs.^8^ However, Stephen *et al*’s comprehensive study highlights that people with FND accrue similar costs to those with refractory epilepsy and demyelinating disorders. The cost of FND alone was estimated to be $1.2 billion annually in the USA in 2017,^9^ and these costs appear to depend on the patient’s satisfaction with the explanation of their diagnosis.^10^ In Denmark, Jennum *et al* showed a nearly tenfold increase in combined direct and indirect costs in FS patients compared with healthy controls.^11^

Studies that assessed indirect costs reported these costs as being higher than the direct medical costs resulting from the disorder.^8^ It has been found that patients with FND are more likely not to be working for health reasons and to be receiving disability-related state financial benefits than patients with other neurological disorders.^12^ No study has yet assessed whether symptom severity and/or duration impact the economic cost of FND.

In this study, we set out to evaluate the direct and indirect costs associated with FND through a retrospective questionnaire-based assessment of people referred to a tertiary FND specialist assessment clinic.

## Methods

### Participants and setting

Participants were patients with scheduled new appointments at St George’s Hospital FND Clinic from 17 October 2017 until 6 February 2018. St George’s Hospital Neurology Department is the regional specialist tertiary neuroscience inpatient and outpatient centre for over 3 million people across South-West London, Surrey and Sussex.

Patients attending the clinic for follow-up appointments and patients with primary diagnoses other than FND were excluded from the study. The diagnosis of FND was made by a neurologist and/or neuropsychiatrist using the criteria outlined in the Diagnostic and Statistical Manual of Mental Disorders (DSM), fifth edition.^13^

### Data and collection

Prior to attending their new appointment, patients were asked to complete a questionnaire. The questionnaire asked participants to retrospectively assess their National Health Service (NHS) resource use, including inpatient, outpatient and community-based care. Patients were also asked to report the effect of FND on their own economic status, for example, any change in employment and/or government benefits received. The study was registered and approved after review as a service evaluation with the clinical governance and audit office at St George’s Hospital. Costs were measured in 2018 Pound Sterling (£)

### Research instruments

#### Client service receipt inventory (CSRI)

A modified version of the CSRI was employed to quantify the health and social care resource use in the 6 months preceding patient consultation (see online supplemental appendix A). The CSRI has been used to quantify health and social care resource use in patients with chronic neurological disorders.^14^ The CSRI was modified and adapted to be more specific to the cohort of patients with FND, based on previous CSRI-included studies^14^ and informed input from specialist consultants in the FND clinic.

10.1136/bmjno-2023-000606.supp1 Supplementary data

Healthcare resource data obtained by the modified CSRI included hospital outpatient appointments, treatments and medications, investigatory procedures, inpatient and residential care, and care provided by all primary and secondary healthcare professionals. Economic and social information included patient employment and informal care received by friends and relatives.

#### EuroQol-five dimension (EQ-5D)

The EQ-5D is a standardised instrument used for measuring generic health status. It is a self-reported scale comprising five dimensions: mobility, self-care, usual activities, pain/discomfort and anxiety/depression. The use of the EQ-5D aimed to investigate the relationship between symptomology and resource use in the cohort, more specifically that of symptom severity with frequency and type of resources used.

#### Patient Health Questionnaire (PHQ-9) and Generalised Anxiety Disorder Scale (GAD-7)

The PHQ-9 is a self-report measure of depression consisting of nine items matching the DSM, fourth edition criteria for major depression. Respondents are asked to rate each of the items on a scale of 0–3 on the basis of how much a symptom has bothered them over the last 2 weeks.^15^ The GAD-7 is a seven-item, self-reported anxiety questionnaire designed to assess the patient’s health status during the previous 2 weeks.^16^ Both of these scales were completed by patients as part of their standard clinical assessment.

### Data analysis

To provide an estimate of the cost of health and social care resource use, the type and frequency of resource use were combined with the national unit costs. National unit costs were extracted from ‘Unit Costs of Health and Social Care 2015’^17^ and supplementary sources.^18 19^ Medication costs were calculated using the information in the British National Formulary.^20^

When accounting for the contact time of the participants with the unit cost for specific healthcare professionals, the patients’ account of contact time was deemed more reliable than the published ‘average consultation time’. This was based on the likelihood that the FND patient group deviated from the mean consultation time of all patients. The complexity of patients with FND requires a multifaceted consultation approach to address not only the physical symptoms but also the psychological and social implications of FND. Therefore, the use of ‘average consultation time’ in this cohort would likely result in inaccurate patient costs.^21^

The participants’ loss of employment income because of their FND was costed based on their employment income before and after symptom presentation, based on data gathered in the CSRI ‘Section 2’. The value of income lost was estimated using national average salaries in line with the participant’s job sector and job title.^22^

The informal care received by the participants was quantified using the replacement cost method,^23^ that is, time spent by friends and relatives providing informal care and assistance was valued as equal to the cost of a paid professional that the friend and/or relative had hypothetically replaced. Therefore, the informal care received was valued at £18 per hour, equal to the Curtis 2015 data on a local authority care worker.^17^

Statistical analysis was performed with the JASP statistic software package. Data were expressed as means±SD. Comparisons between groups were performed with the analysis of non-parametric tests. A value of p<0.05 was considered statistically significant.

## Results

### Study demographics

Questionnaires were sent to 328 patients and completed by 118 participants, with a response rate of 36%. 83 questionnaires had every section completed. Most patients identified as white British (77%), followed by black Africans and black Caribbeans (6% each).

### Direct health costs

The breakdown of costs by service is given in tables 1 and 2. Despite being used by only 6.36% of patients, the cost associated with intensive care unit admissions had the highest mean cost per patient at £629.15. This was followed by neurology ward admissions (also used by only 6% of patients), which carried a mean cost of £245.38. GP consultations, whether in person (81% of patients) or by phone (46% of patients), were used by most patients and carried a mean cost per patient of £245.

**Table 1:** Inpatient service utilisation and cost in the 6 months prior to new appointments (n=110)

Service	Count*	Duration of resource utilisation (days/visits)		Cost of resource utilisation (£)	
	N (%)	Total cohort contact time	Mean contact time	Total cohort cost	Mean cost (SD)
Intensive care unit	7 (6.4%)	59	0.54	69 207	629 (3418)
Medical inpatient ward	19 (17.3%)	140.5	1.28	67 721	615 (1883)
Neurology inpatient ward	7 (6.4%)	56	0.51	26 992	245 (1247)
Accident and emergency	38 (34.6%)	162	1.47	22 356	203 (548)
Other inpatient Wards	4 (3.6%)	29	0.26	13 978	127 (923)
Assessment/rehab ward	8 (7.3%)	19.5	0.18	12 051	110 (480)
Day unit/investigation unit	5 (4.6%)	7	0.06	3374	31 (150)

		Total number of investigations	Number of investigations per patient		
MRI scan of head or back	43 (39.1%)	55	0.50	8030	73 (108)
CT scan of head	25 (22.7%)	31	0.28	3069	28 (57)
Nerve conduction study	19 (17.3%)	20	0.18	2760	25 (57)
Electroencephalography	17 (15.5%)	20	0.18	2760	25 (65)
Lumbar puncture	6 (5.5%)	7	0.06	1428	13 (57)
Total				233 726	**2124.78**

*Count and percentage of the cohort that used this service.

**Table 2:** Outpatient service utilisation and cost in the 6 months prior to new appointments (n=110)

Service	Count *	Duration of resource utilisation (Minutes)		Cost of resource utilisation (£)	
	N (%)	Total contact cohort time	Mean contact time	Total cohort cost	Mean cost (SD)
GP—surgery	89 (80.9%)	6121	55.65	20 199.30	183.63 (197)
GP—phone	51 (46.4%)	1846.5	16.79	6739.73	61.27 (134)
Psychiatrist	19 (17.3%)	4570	41.55	8043.20	73.12 (315)
Neurologist	69 (62.7%)	3280	29.82	5674.40	51.59 (62)
Other doctor	44 (40%)	5451	49.55	6050.61	55.01 (149)
Physiotherapist—hospital	25 (22.7%)	3200	29.09	2912.00	26.47 (76)
Physiotherapist—home	18 (16.4%)	3080	28.00	2802.80	25.48 (96)
Dentist	36 (32.7%)	1348.5	12.26	2723.97	24.76 (62)
Psychologist	12 (10.9%)	2375	21.59	2161.25	19.65 (89)
Nurse specialist	15 (13.6%)	1185	10.77	2133.00	19.39 (77)
GP—home	13 (11.8%)	490	4.45	1617.00	14.7 (47)
Social worker visit	15 (13.6%)	1545	14.05	1421.40	12.92 (55)
Occupational therapist—home	20 (18.2%)	1975	17.95	1323.25	12.03 (36)
General practice nurse	27 (24.6%)	1391	12.65	834.60	7.59 (30)
Other nurse or therapist	6 (5.5%)	650	5.91	526.50	4.79 (23)
Social worker phoned	11 (10%)	480	4.36	441.60	4.01 (15)
Physiotherapist—GP or clinic	4 (3.6%)	380	3.45	326.80	2.97 (18)
Mental health worker	8 (7.3%)	400	3.64	252.00	2.29 (9)
Occupational therapist—hospital	6 (5.5%)	335	3.05	251.25	2.28 (11)
Speech therapist	4 (3.6%)	75	0.68	54.75	0.50 (3)
Medication	80 (67.8%)			29 526.19	268.42 (986)
Total				96 015.6	872.87

*Count and percentage of the cohort that used this service.

Of note, patients who were depressed (defined as having a PHQ-9 score of >10) incurred greater mean costs to the NHS than those patients who were defined as not depressed (£4380 vs £1503, t(83)=−3.25, p<0.001). The same phenomenon was true for patients defined as anxious (GAD-7 score of >10) versus those defined as not anxious (£4017 vs £1980, t(82)=−2.1, p<0.001).

Many participants incurred substantial out-of-pocket expenses in the form of adaptations made to residences for the purpose of disabled access. The mean out-of-pocket expense of these participants who made modifications was £3499.47 (±£5299.60), while the mean across the full cohort was £570.85 (±£2446.71).

Home-based services are summarised in table 3. A small minority of patients used these services, which perhaps highlights the skewed distribution of the health-resource use of patients with FND.

**Table 3:** Utilisation of home-based services and cost in the 6 months prior to new appointments (n=110)

Service	Count*	Duration of resource utilisation (min)		Cost of resource utilisation (£)	
	N (%)	Total cohort contact time	Mean contact time	Total cohort cost	Mean cost (SD)
Help with personal care†	9 (8.2%)	55 131.5	501.20	18 193.40	165.39 (771)
Domestic help†	4 (3.6%)	19 560	177.82	6454.80	58.68 (409)
Qualified nurse (eg, district nurse)	7 (6.4%)	576	5.24	420.48	3.82 (19)
Transport to healthcare appointments‡	7 (6.4%)	–	–	390.00	3.55 (14)

*Count and percentage of the cohort that used this service.

†Social services funded.

‡National Health Service funded.

**Figure 1:**
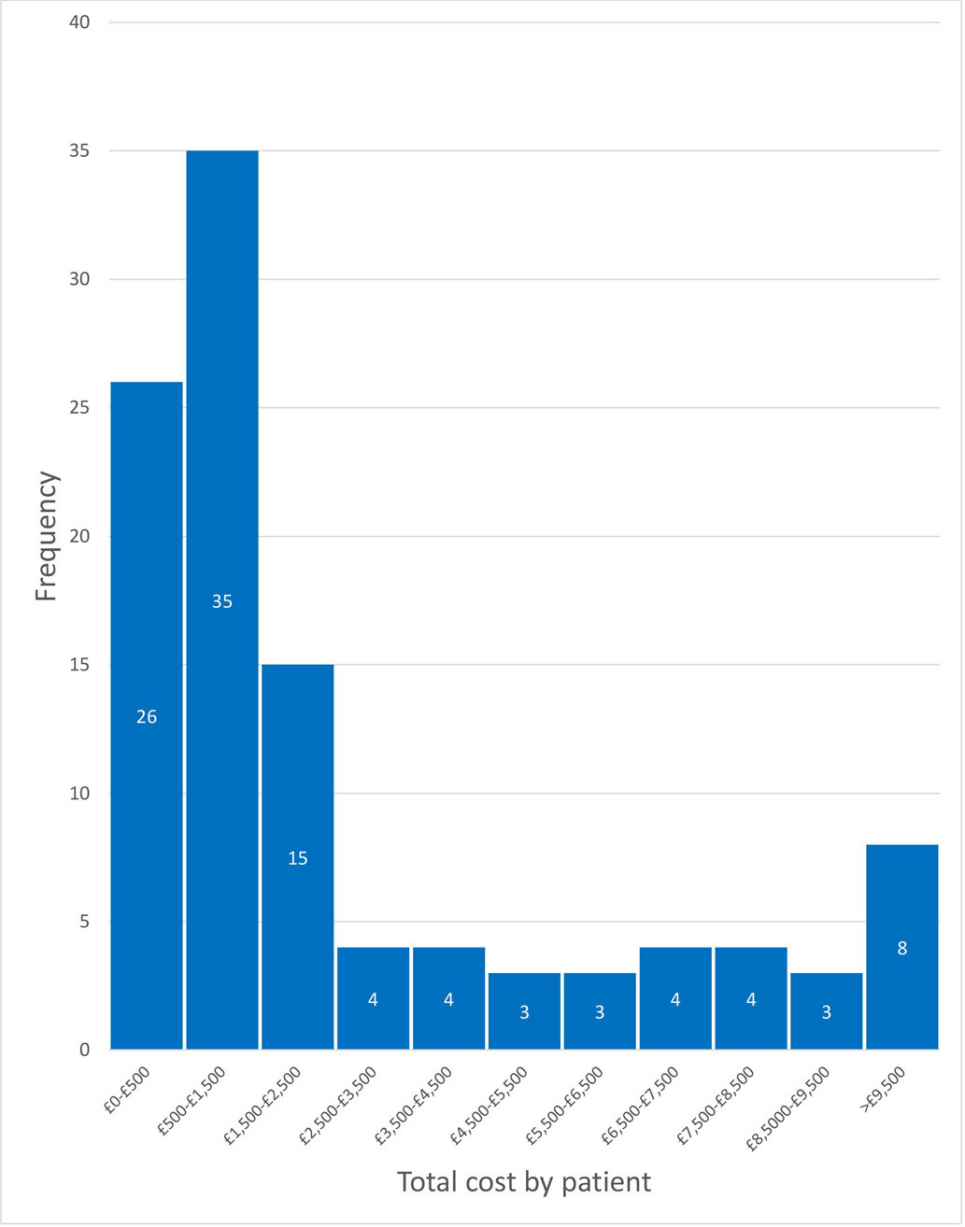
Costs to National Health Service by patient in the 6 months prior to new appointments.

Total costs to the NHS per patient are displayed in figure 1. These costs include all costs listed in tables 1 and 2, as well as transport to NHS appointments and visits by the district nurse. The mean cost per patient was £3229 (±5,395.93), with a median value of resource use of £1152.27. 12.68% of respondents reported costs of over £5000, predominantly due to inpatient admission.

### Indirect costs

**Figure 2:**
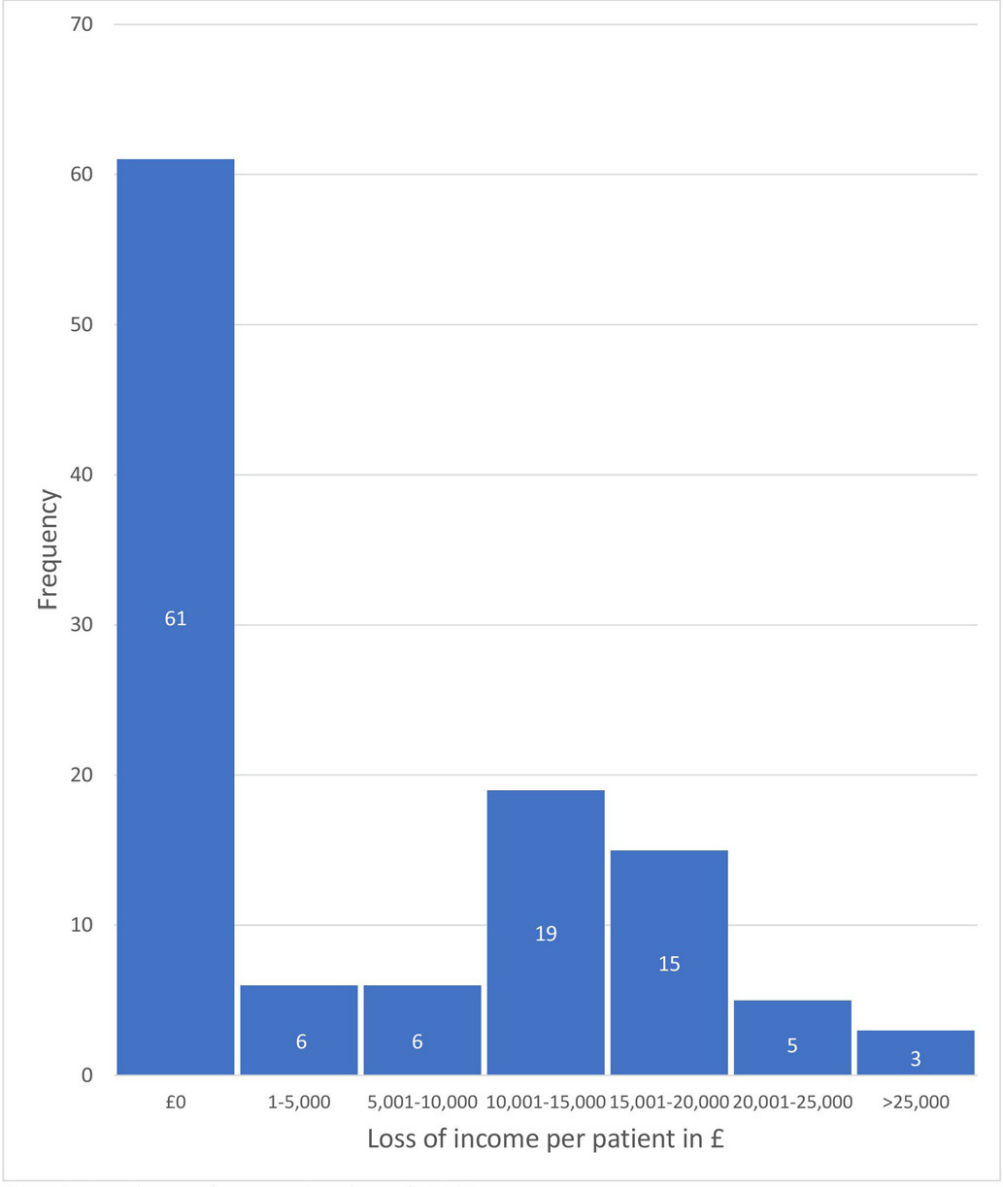
Annual income loss per patient due to their functional neurological disorder.

There was a substantial cost of lost income in the cohort, which was calculated as estimated annual income prior to the onset of their FND, less annual income after onset (table 4), estimated at a total of £758 355 among 115 patients. This represents a mean of £6594.4 (±8503) among all patients. Excluding participants who were unemployed prior to symptom onset, the mean loss of income was £10 821.91 (±8306). The amount of income lost per patient is shown in figure 2.

**Table 4:** Change in employment status due to their FND (n=115)

Employment status	Before FND	Currently
	No. (%)	No. (%)
Employed full time	54 (47%)	19 (16.5%)
Employed full time ‘off sick’	1 (0.9%)	6 (5.2%)
Employed part-time	12 (10.4%)	6 (5.2%)
Employed part-time ‘off sick’	1 (0.9%)	2 (1.7%)
Unemployed	13 (11.3%)	43 (37.4%)
Self-employed	5 (4.4%)	5 (4.4%)
Self-employed ‘off sick’	2 (1.7%)	3 (2.6%)
Retired (because of age)	3 (2.6%)	6 (5.2%)
Retired (because of ill health)	5 (4.4%)	10 (8.7%)
Student	4 (3.5%)	5 (4.4%)
Student—interrupted due to health	7 (6.1%)	1 (0.9%)
Housewife/husband	6 (5.2%)	5 (4.4%)
Other	2 (1.7%)	4 (3.5%)

FND, functional neurological disorder.

Only 16.5% of study participants were able to maintain full-time employment, with another 5.2% employed on a part-time basis. Of the 54 and 12 people in full-time and part-time employment before the onset of FND, only 19 (35%) and 6 (50%) people, respectively, remained in employment. With their lack of income from employment, many patients became reliant on government benefits to supplement or replace their income. Of the cohort, 71.8% received welfare benefits over the preceding 6 months, with the mean amount received being £299.50 (±180.76) per week.

Loss of productivity affected not only patients but also their carers, friends and family, as shown in table 5. Patients estimated receiving a mean of almost 20 hours per week (median 13.75 hours per week) of informal care.

**Table 5:** Weekly hours and cost of informal care (n=100)

Informal care method	Mean hours spent per week (SD)	Mean estimated value per week (SD)	Mean hours spent per 6 months (SD)	Mean estimated value per 6 months (SD)
Personal care	3.16 (5.64)	56.88 (102)	82.16 (146.64)	1478.88 (2652)
Housework	4.1 (6.12)	74.09 (110)	107.02 (159.12)	1926.41 (2860)
Transport	2.92 (3.58)	52.515 (65)	75.86 (93.08)	1365.39 (1690)
Preparing meals	3.46 (4.96)	62.28 (89)	89.96 (128.96)	1619.28 (2314)
Gardening	0.71 (1.25)	12.83 (23)	18.53 (32.5)	333.45 (598)
Shopping	1.72 (2.28)	30.96 (41)	44.72 (59.28)	804.6 (1066)
Looking after pets	1.63 (3.87)	29.34 (70)	42.38 (100.62)	762.84 (1820)
Home improvements	0.81 (1.68)	14.58 (30)	21.06 (43.68)	379.08 (780)
Other	1.25 (8.16)	22.5 (147)	32.5 (212.16)	585 (3822)
Total informal care	19.78 (21.89)	355.97 (394)	514.28 (596.14)	9255.29 (10242.27)

### Total health costs

The total health costs of the cohort are shown in table 6. Total costs were also positively skewed, with a skewness value of 0.78 and a kurtosis value of −0.719.

**Table 6:** Summary of 6-month costs by service type (n=118)

	Mean* (SD)	Median*	Range
Inpatient service use	1960.72 (4560.89)	0	0–33 396
Outpatient	604.45 (596.56)	431.9	0–3015
Medication	268.42 (986.30)	50.1	0–10 089.64
Home-based services	231.44 (876.02)	0	0–4752
Diagnostic investigations	164.06 (211.56)	138	0–1196
Total service cost	3229.09 (5395.93)	1117.54	0–34 915.64
Employment lost	6594.40 (8503.74)	0	0–30 048
Informal care	9255.29 (10 242.27)	6435	0–41 184
Total indirect cost	15 840.69 (17 889.74)	6828	0–65 204
Adaptations	570.85 (2446.71)	0	0–18,200
Total costs	19 649.63 (22 134.54)	9334.65	180–76 729.86

### Intangible costs

**Figure 3:**
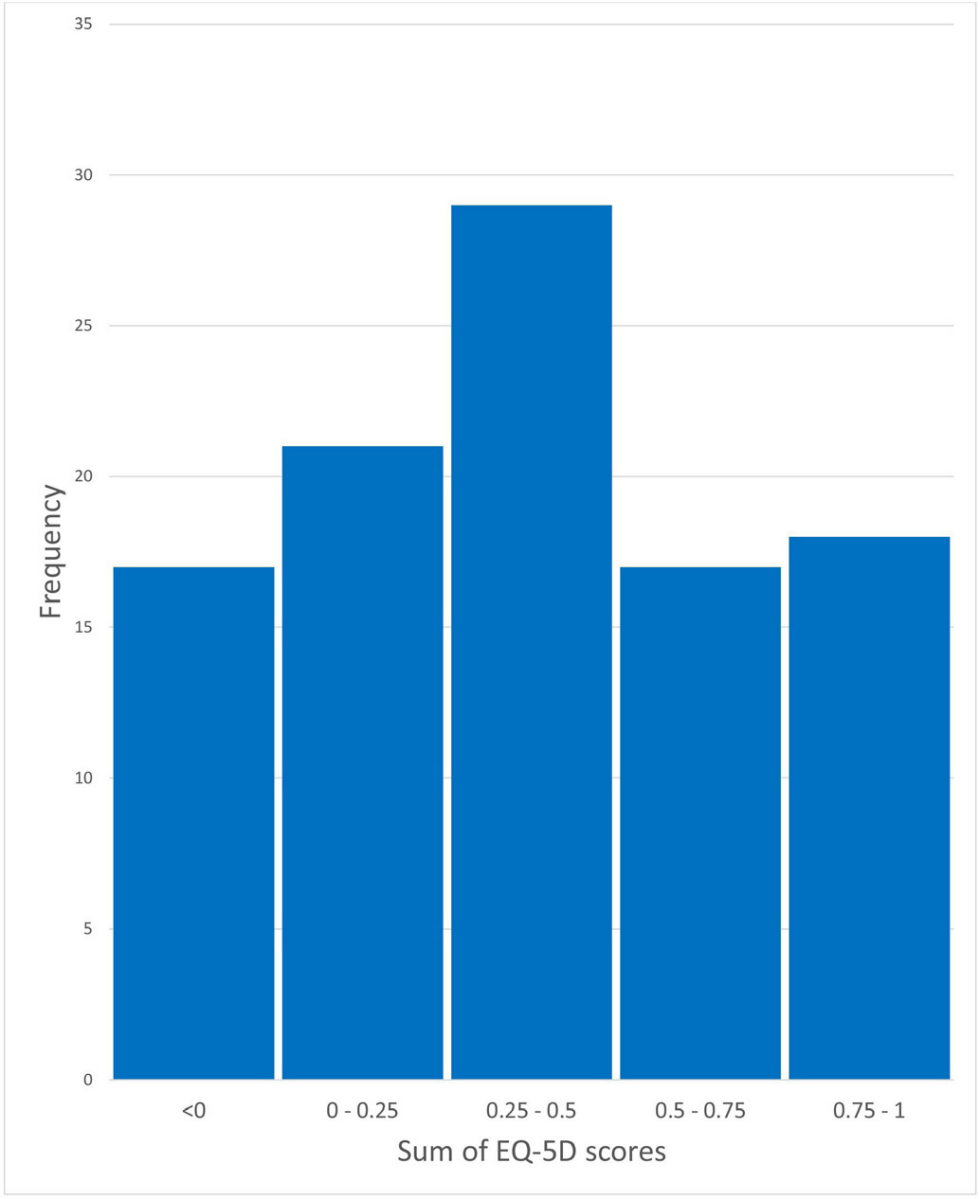
Distribution of total EQ-5D scores by patient. EQ-5D, EuroQoL-five dimension.

The distribution of EQ-5D scores is shown in figure 3. There was no significant relationship between the duration of the disorder and EQ-5D score (p=0.36, Spearman’s r=0.13). After the removal of outliers, defined as >3 SD from the mean, there was a significant relationship between the duration of symptoms and total cost to NHS in the prior 6 months (p=0.04, Spearman’s r=0.226). There was no significant relationship between the severity of symptoms and total cost to NHS in the prior 6 months (p=0.063, Spearman’s r=0.218).

## Discussion

This study highlights the high healthcare costs of patients with FND. These patients were found to have a mean utilisation of health resources valued at £3229 over the 6-month period (£6458 p/a) prior to their initial appointment at the tertiary neuropsychiatry service. Extrapolation of this mean value using an estimated incidence of 4–12 per 100 000 per year^7 24 25^ gives a total cost of NHS resource use of between £13.5 million and £40.4 million per year. This estimate is nearly eight times that of the national health expenditure per person, almost twice that of estimates of the cost per patient of chronic obstructive airway disease (£3488 p/a) and almost four times that of depression (£1873 p/a) and diabetes (1870 p/a).^26^ Such comparisons are, however, limited by heterogeneous methods of cost estimation. Jennum *et al* 11 compared the cost of patients with FS to age-matched and location-matched controls and found direct healthcare costs to be 4.8 times greater in the FS group. Both findings demonstrate the high direct healthcare costs of people with FND.

The distribution of these direct costs was positively skewed, resulting in a small number of patients requiring the use of costly interventions, including admissions to hospitals and intensive care units. The most frequently used services were outpatient services, particularly GPs. However, as shown in tables 1 and 2, the most costly resource utilisations in the cohort were, in decreasing order, admission to an intensive care unit, admission to a medical in-patient ward, GP appointments and emergency department visits.

As in other cost of illness analyses, it is difficult to isolate the ‘pure’ cost of FND, that is, the cost that does not stem from any comorbid conditions. Any cost estimates reported in this study represent the yearly direct and indirect costs of patients with FND. Attempting to assess such a pure cost may be an exercise in futility, given the nature of the interaction of FND with its psychiatric comorbidities. Whether FND symptom severity and outcome are positively or negatively affected by a mood or anxiety disorder is unclear.^27 28^ Our findings suggest that, in any case, symptom severity is not correlated with higher health costs. Our findings of increased costs for patients with FND who also suffer from depression and anxiety may indicate only an added cost of these two disorders, which has been described in the literature,^29 30^ or they may point to a synergistic relationship. The investigation of this question is beyond the scope of a self-reported, retrospective review but may offer an avenue for future research.

As is the case in previous studies that investigated the indirect costs of FND,^8^ our findings show that the indirect costs of the disorder dwarf the already considerable direct costs. Total indirect costs per patients were a mean of £15 850. Such indirect costs are borne by both patients and their family or friends, as well as by taxpayers, in supporting patients who are no longer able to gain money from employment. Such high indirect costs are compounded by patients with FND having worse outcomes when in receipt of government welfare.^31^

Comparing our findings to the literature on the economic costs of FND is challenging given the geographical, clinical and methodological heterogeneity of the studies in this area. Looking specifically at studies investigating adults with FND in countries with a similar public healthcare system, in patients with psychogenic non-epileptic seizures (PNES), Goldstein *et al* 32 in the UK found similar healthcare utilisation costs (£3943), but substantially lower productivity loss (£2953) in a cohort of n=367 in the 6 months prior to treatment. Magee *et al* 33 in Ireland assessed the cost of PNES to taxpayers and reported direct costs of €2714.5 per 6 months, with combined social welfare payments and loss of tax revenue costs calculated at €7783 per 6 months per person. Deleuran *et al* in Denmark found direct healthcare costs of €2904 over 6 months in patients with FS.^34^ Finally, Tinazzi *et al* in Italy reported an average direct hospital cost of €1652 per 6 months for patients referred to a specialist functional movement disorder clinic.^35^

### Implications for clinical practice

An important finding from the literature relating to treatment interventions in FND is a decline in healthcare resource utilisation^36–38^ and economic cost following an intervention, whether this is psychotherapy, structured delivery of a robust diagnosis or specialist physiotherapy.^8^ Our findings suggest that patients with a longer duration of FND continue to have higher costs in the months prior to diagnosis than those with a shorter duration. Additionally, our findings suggest that it is incorrect to assume that the correlation between NHS resource use and duration of disorder is a result of reduced quality of life. This highlights the potential cost savings of early intervention to minimise monetary and quality of life costs for both the patient and society. An important first step would be to increase patient access to specialist services and/or to improve general knowledge of the condition. Referral to a specialist in FND may reduce the latency to diagnosis and avoid unnecessary consultations and tests.^7^ This study investigated only costs in the 6 months prior to the patients’ attendance at the FND clinic and costs subsequent to attendance and diagnosis should be studied to investigate any change.

## Limitations

We acknowledge some study limitations. First, the use of the patient-reported CSRI is liable to recall bias. While this limitation should be considered, the results of a 2005 study suggest that retrospective self-report data can be equally reliable as medical database data.^39^ Also of note, the data gathered on participants’ medication use through self-reporting had a surprisingly low completion rate. Rather than this signifying that fewer patients than expected were taking medication, it could be that participants did not complete this section due to a lack of knowledge of the names of their medications. Therefore, one can argue the data on medication should be treated as a minimum possible value.

A second limitation is the low return rate of the questionnaire, resulting in a relatively small sample size. Furthermore, the large number of questionnaire non-responders (63.99% of the intended cohort) could signify a selection bias in the study, limiting the external validity of these findings.

Third, the patients included in our study are those referred to a tertiary specialist service, and as such, they likely represent a severely affected cohort. Such referral bias would also limit the external validity of the study’s results.

Fourth, our study would ideally have used a comparator group so that the costs associated with FND could have been contextualised. Failing the use of a comparison with healthy controls or another neurological disorder, one possibility might have been to compare the costs of patients before and after their diagnosis of FND, as there has yet to be a study comparing indirect costs before and after patients receive a diagnosis of FND. This is an ongoing project.

Finally, the costs estimated in this study may differ and be lower than those in other countries, such as Australia^40^ and the USA,^41^ where unit costs may differ for varying aspects of healthcare.

### Conclusions

This study highlights the high cost of FND for both patients and the NHS. Patients with a longer duration of suffering from FND were shown to have higher direct and indirect costs than those with a shorter duration of the disorder. Our findings are consistent with similar studies’ reporting the high direct costs and higher indirect costs of the disorder. Adequate reform of the patient pathway and reorganisation of NHS services to make diagnoses and initiate treatment more quickly would likely reduce these costs.

## Data Availability

Data are available upon reasonable request. Requests for data can be sent to brianw.omahony@gmail.com.
