# Supplementary material for: Cross-sectional evaluation of health resource use in patients with functional neurological disorders referred to a tertiary neuroscience centre
Source: BMJ Neurol Open. 2024 May 22;6(1):e000606. doi: 10.1136/bmjno-2023-000606 (PMC11116875; doi:10.1136/bmjno-2023-000606)
Supplement: Supplementary data [file bmjno-2023-000606supp001.pdf]

Appendix: C. The Modified Client Service Receipt Inventory (CSRI)

□□□□□ : □□

Neurology Service
Background Information Questionnaire
Professor Mark Edwards

For Office-Use only
Initials: □□ ***Hosp No.*** **……………………**

Dear Sir/Madam,

You are due to be seen in the Neurology Clinic at St George's Hospital, London in the near future. We would like to gather some information about how the problem(s) you are experiencing affect your life. We would be grateful if you could fill in the following questionnaires before your appointment to help us understand.

Please try to answer every question. You may find that some questions are not relevant to your problem, but we would be grateful if you try to answer the question anyway.

Please bring the completed questionnaires to your neurology appointment.

Completing these questionnaires is entirely voluntary, and it will not affect the care you receive if you choose not to complete them. We are routinely collecting this information from all new patients we see.

By completing these questionnaires, you are consenting for your anonymised answers to be used to help us to understand the impact your neurological problems and symptoms have had on your life. Your responses will help us to provide you with better care, and help us to develop the services we offer patients. We will collate this information and may publish findings in the scientific literature. All your responses on these questionnaires will be **anonymised and kept confidential**, and you would not be identifiable in any published studies.

If you would like help to complete the questionnaires, we can arrange this after the appointment with the doctor.

Thank you.

Page **1** of **17**

☐☐☐☐☐: ☐☐

For Office-Use only
Initials: ☐☐ *Hosp No.* ....

Date Completed: ☐☐ ☐☐ ☐☐☐☐
Date Month Year

General Information Questionnaire

Section 1: Yourself:

Please indicate the ethnic group you identify with:

White	British	1. ☐	Asian	Indian	7. ☐
White	Irish	2. ☐	Asian	Pakistani	8. ☐
White	Other	3. ☐	Asian	Bangladeshi	9. ☐
Black	Caribbean	4. ☐	Asian	Other	10. ☐
Black	African	5. ☐	Chinese		11. ☐
Black	Other	6. ☐		Other.. (please state)	12. ☐

When did your neurological disorder begin? ☐☐ ☐☐ ☐☐☐☐
(state year if the exact date is unknown) Date Month Year

Page **2** of **17**

☐☐☐☐☐: ☐☐

Occupational and financial Information

We are asking you the following questions because we would like to know the impact of your neurological disorder in economic terms to you and to society in general

Section 2: Your employment

What was <u>your</u> employment status <u>before</u> you developed your disorder?

Employed full-time and working	1. ☐	Retired (because of age)	8. ☐
Employed full-time but 'off-sick'	2. ☐	Retired (because of ill health)	9. ☐
Employed part-time	3. ☐	Student	10. ☐
Employed part-time but 'off-sick'	4. ☐	Student - interrupted due to illness	11. ☐
Unemployed	5. ☐	Housewife/Househusband	12. ☐
Self-employed and working	6. ☐	Other	13. ☐
Self-employed but 'off-sick'	7. ☐	(please state)	

If you worked, how many hours per week did you work at that time? ....

If you worked, have you had to stop or reduce work/study because of your disorder? 1. ☐ Yes 2. ☐ No

If yes to the above, how many hours a week are you currently working? ....

<u>If you worked</u>, what was your job sector?

Accountancy, banking and finance	1. ☐	Law enforcement and security	10. ☐
Business, consulting and management	2. ☐	Marketing, advertising and PR	11. ☐
Creative arts and design	3. ☐	Property and construction	12. ☐
Engineering and manufacturing	4. ☐	Public services and administration	13. ☐
Environment and agriculture	5. ☐	Recruitment and HR	14. ☐
Healthcare	6. ☐	Retail	15. ☐
Hospitality and events management	7. ☐	Science and pharmaceuticals	16. ☐
Information technology	8. ☐	Social care	17. ☐
Law	9. ☐	Leisure, sport and tourism	18. ☐

<u>If you worked</u> before you developed your disorder, what was your job title?

....

What is <u>your current</u> employment status?

Employed full-time and working	1. ☐	Retired (because of age)	8. ☐
Employed full-time but 'off-sick'	2. ☐	Retired (because of functional neurological disorder)	9. ☐
Employed part-time	3. ☐	Retired (because of other illness)	10. ☐
Employed part-time but 'off-sick'	4. ☐	Student	11. ☐
Unemployed	5. ☐	Student - interrupted due to disorder	12. ☐
Self-employed and working	6. ☐	Housewife/husband	13. ☐
Self-employed but 'off-sick'	7. ☐	Other	14. ☐

Page **3** of **17**

☐☐☐☐☐:☐☐

If you are currently working, what is your job sector?

Accountancy, banking and finance	1. ☐	Law enforcement and security	10. ☐
Business, consulting and management	2. ☐	Marketing, advertising and PR	11. ☐
Creative arts and design	3. ☐	Property and construction	12. ☐
Engineering and manufacturing	4. ☐	Public services and administration	13. ☐
Environment and agriculture	5. ☐	Recruitment and HR	14. ☐
Healthcare	6. ☐	Retail	15. ☐
Hospitality and events management	7. ☐	Science and pharmaceuticals	16. ☐
Information technology	8. ☐	Social care	17. ☐
Law	9. ☐	Leisure, sport and tourism	18. ☐

If you are currently working, what is your job title?

……………………………………………………………………………………………………

If you are currently working, have you taken any time off work in the last 6 months because of your health ?

1. ☐ Yes 2. ☐ No 3. ☐ I have not worked in the past 6 months

If yes please give details below: *(Please tell us either the number of day or the number of hours you took off in the last 6 months)*

	Yes	NO	Number of Days	Number of hours
Took sick leave from work	☐	☐	………….	………….
Used your paid holiday time from work	☐	☐	………….	………….
Took unpaid leave	☐	☐	………….	………….
Just made up the time at work	☐	☐	………….	………….
Other arrangements (please describe)	☐	☐	………….	………….

………………………………………………..

Section 3: Benefits

What benefits do you currently receive? Please tick as many as are applicable.

Do not receive any benefits	1. ☐	Industrial injuries disablement benefit	18. ☐
Attendance allowance	2. ☐	Maternity allowance	19. ☐
Bereavement Allowance	3. ☐	Jobseekers allowance	20. ☐
Carer's allowance	4. ☐	Pension credit	21. ☐
Child benefit	5. ☐	Personal independence payment (High rate)	22. ☐
Constant attendance benefit	6. ☐	Personal independence payment (Low rate)	23. ☐
Disability living allowance	7. ☐	Reduced earning allowance	24. ☐
Disablement benefit	8. ☐	Retirement allowance	25. ☐
Employment and support allowance (Mobility - high rate)	9. ☐	Severe disability allowance	26. ☐
Employment and support allowance (Mobility - low rate)	10. ☐	State retirement pension	27. ☐

Page **4** of **17**

☐☐☐☐☐:☐☐

Employment and support allowance (Daily Living - high rate)	11. ☐	Statutory sick pay	28. ☐
Employment and support allowance (Daily Living - low rate)	12. ☐	Universal credit	29. ☐
Exceptionally severe disablement allowance	13. ☐	Widowers pension	30. ☐
Guardians allowance	14. ☐	Widowed parents allowance	31. ☐
Housing benefit	15. ☐	Winter fuel payment	32. ☐
Incapacity benefit	16. ☐	Working and child tax credits	33. ☐
Income support	17. ☐	Other………………………………	34. ☐

Health and Social Services received

Section 4: Out-patient services received

This section is for you to record information about the face-to-face out-patient consultations you have had with professionals in the last 6 months- not including today's appointment

Care provider	Have you used this service?	Number of visits in the last 6 months	How long did the average visit last?
**A. General practitioner-The Surgery**	1. ☐ Yes 2. ☐ No	…………….	………………… Minutes
**B. General practitioner- At home**	1. ☐ Yes 2. ☐ No	…………….	………………… Minutes
**C. General practitioner- Phone**	1. ☐ Yes 2. ☐ No	…………….	………………… Minutes
**D. Neurologist**	1. ☐ Yes 2. ☐ No	…………….	………………… Minutes
**E. Psychiatrist**	1. ☐ Yes 2. ☐ No	…………….	………………… Minutes
**F. Other doctor (state type)** ………………………………	1. ☐ Yes 2. ☐ No	…………….	………………… Minutes
**G. Other doctor (state type)** ………………………………	1. ☐ Yes 2. ☐ No	…………….	………………… Minutes
**Y. Other doctor (state type)** ………………………………	1. ☐ Yes 2. ☐ No	…………….	………………… Minutes
**Z. Other doctor (state type)** ………………………………	1. ☐ Yes 2. ☐ No	…………….	………………… Minutes
**H. General practice nurse**	1. ☐ Yes 2. ☐ No	…………….	………………… Minutes
**I. Nurse specialist**	1. ☐ Yes 2. ☐ No	…………….	………………… Minutes

Page **5** of **17**

☐☐☐☐☐:☐☐

**J. Physiotherapist- In Hospital**	1. ☐ Yes	2. ☐ No	....	.... Minutes
**K. Physiotherapist- Home**	1. ☐ Yes	2. ☐ No	....	.... Minutes
**L. Physiotherapist- At the GP**	1. ☐ Yes	2. ☐ No	....	.... Minutes
**M. Occupational therapist- Hospital**	1. ☐ Yes	2. ☐ No	....	.... Minutes
**N. Occupational therapist-At the GP**	1. ☐ Yes	2. ☐ No	....	.... Minutes
**O. Occupational therapist- At home**	1. ☐ Yes	2. ☐ No	....	.... Minutes
**P. Speech therapist**	1. ☐ Yes	2. ☐ No	....	.... Minutes
**Q. Other nurse or therapist** …………………………....	1. ☐ Yes	2. ☐ No	....	.... Minutes
**R. Psychologist**	1. ☐ Yes	2. ☐ No	....	.... Minutes
**S. Mental health worker**	1. ☐ Yes	2. ☐ No	....	.... Minutes
**T. Dentist**	1. ☐ Yes	2. ☐ No	....	.... Minutes
**U. Other (please state) *** …………………………....	1. ☐ Yes	2. ☐ No	....	.... Minutes
**V. Other (please state) ***	1. ☐ Yes	2. ☐ No	....	.... Minutes
**W. Social worker- Visit**	1. ☐ Yes	2. ☐ No	....	.... Minutes
**X. Social worker- Phoned**	1. ☐ Yes	2. ☐ No	....	.... Minutes

Section 5: In-patient, residential and/or day care received

In the last 6 months, have you attended A&E or have you been a hospital in-patient? If yes, please give the total number of attendances or total length of stay in the last 6 months.

**A. Intensive care unit**	1. ☐ Yes	2. ☐ No	.... Weeks .... Days
**B. Neurology in-patient ward**	1. ☐ Yes	2. ☐ No	.... Weeks .... Days
**C. Medical in-patient ward**	1. ☐ Yes	2. ☐ No	.... Weeks .... Days
**D. Assessment/rehabilitation ward**	1. ☐ Yes	2. ☐ No	.... Weeks .... Days

Page **6** of **17**

☐☐☐☐☐:☐☐

**E. Other in-patient ward (state type)** …………………………....	1. ☐ Yes	2. ☐ No	.... Weeks .... Days
**F. A&E Attendances** …………………………....	1. ☐ Yes	2. ☐ No	.... Number of attendances
**N. Calling an ambulance** …………………………....	1. ☐ Yes	2. ☐ No	.... Number of times

In the last 6 months, have you spent time in any day care setting (e.g. did not stay overnight)? Please give the average number of attendances per month.

**G. Day unit / investigation unit**	1. ☐ Yes	2. ☐ No	.... Attendances per month
**H. NHS Day care**	1. ☐ Yes	2. ☐ No	.... Attendances per month
**I. Social services day centre**	1. ☐ Yes	2. ☐ No	.... Attendances per month
**J. Voluntary organisation day centre**	1. ☐ Yes	2. ☐ No	.... Attendances per month
**K. Support groups or societies**	1. ☐ Yes	2. ☐ No	.... Attendances per month
**L. Other day setting (please describe)** …………………………....	1. ☐ Yes	2. ☐ No	.... Attendances per month
**M. Other day setting (please describe)** …………………………....	1 ☐ Yes	2. ☐ No	.... Attendances per month

Section 6: Home based services received

In the last six months have you had any home nursing services? Please fill in the average number of visits received per month and how long each visit lasted.

**A. Qualified nurse (e.g. district nurse) social services funded**	1. ☐ Yes	2. ☐ No	.... per month	...... hours ...... mins
**B. Qualified nurse privately funded**	1. ☐ Yes	2. ☐ No	.... per month	...... hours ...... mins
**C. Help with personal care (e.g. washing) social services funded**	1. ☐ Yes	2. ☐ No	.... per month	...... hours ...... mins
**D. Help with personal care privately funded**	1. ☐ Yes	2. ☐ No	.... per month	...... hours ...... mins

In the last six months, have you had any domestic services? Please fill in the average number of visits received per month and how long each lasted.

Page **7** of **17**

☐☐☐☐☐: ☐☐

**E. Domestic help social services Funded (e.g. Cooking/Cleaning/Shopping)**	1. ☐ Yes 2. ☐ No	.... per month	...... hours ...... mins	
**F. Domestic help privately funded (e.g. Cooking/Cleaning/Shopping)**	1. ☐ Yes 2. ☐ No	.... per month	...... hours ...... mins	
**G. Meals on wheels**	1. ☐ Yes 2. ☐ No	.... per month	N/A	

In the last six months, have you had any home therapy services? Please fill in the total number of visits received in the last 6 months and how long each lasted on average.

**H. Physiotherapy (NHS)**	1. ☐ Yes 2. ☐ No	.... visits	...... hours ...... mins
**I. Physiotherapy (Private)**	1. ☐ Yes 2. ☐ No	.... visits	...... hours ...... mins
**J. Occupational therapy (NHS)**	1. ☐ Yes 2. ☐ No	.... visits	...... hours ...... mins
**K. Occupational therapy (Private)**	1. ☐ Yes 2. ☐ No	.... visits	...... hours ...... mins
**L. Speech therapy (NHS)**	1. ☐ Yes 2. ☐ No	.... visits	...... hours ...... mins
**M. Speech therapy (Private)**	1. ☐ Yes 2. ☐ No	.... visits	...... hours ...... mins
**N. Other service (please state)**	1. ☐ Yes 2. ☐ No	.... visits	...... hours ...... mins
**O. Other service (please state)**	1. ☐ Yes 2. ☐ No	.... visits	...... hours ...... mins

In the last six months, have you had any other assistance in daily life? Please fill in the average cost per month or total

**P. Transport to healthcare appointments (Via NHS)**	1. ☐ Yes 2. ☐ No		
**P. Transport to healthcare appointments (Privately Funded)**	1. ☐ Yes 2. ☐ No		£....... per month
**Q. Transport to self-help groups (NHS)**	1. ☐ Yes 2. ☐ No		
**Q. Transport to self-help groups (Privately Funded)**	1. ☐ Yes 2. ☐ No		£....... per month
**R. Changes to your home (e.g. stair lift)**	1. ☐ Yes 2. ☐ No	Describe	....

Page **8** of **17**

☐☐☐☐☐: ☐☐

**S. Special equipment (e.g. Kitchen Equipment)**	1. ☐ Yes 2. ☐ No	Describe	....
**T. Financial support (not including state benefits)**	1. ☐ Yes 2. ☐ No		£....... per month

Section 7: Medicine

In the last 6 months, what medicines have you used?

Drug Name (generic or brand)	Date you started taking the Drug	Date you finished (put N/A if you are still using it)

Page **9** of **17**

☐☐☐☐☐: ☐☐

Section 8: Investigations related to your disorder received by you

Please record information about the investigations you have had for your disorder in the last 6 months.

Investigation Type	Have you used this service?	Number of times in the last 6 months
**A. CT scan of head**	1. ☐ Yes 2. ☐ No	....
**B. MRI scan of head or back**	1. ☐ Yes 2. ☐ No	....
**C. Nerve conduction study (NCS/EMG) [Nerve tests]**	1. ☐ Yes 2. ☐ No	....
**D. EEG (brainwave recording)**	1. ☐ Yes 2. ☐ No	....
**E. Lumbar puncture (fluid taken from back)**	1. ☐ Yes 2. ☐ No	....
**F. Video-telemetry (brainwave and video recording as in-patient)**	1. ☐ Yes 2. ☐ No	....
**G. Neuropsychological testing (testing of memory and other brain functions)**	1. ☐ Yes 2. ☐ No	....

☐☐☐☐☐: ☐☐

Section 9: Friends and relatives

In the last 6 months, have friends or relatives helped you with tasks at home which you couldn't do? Please fill in the average number of hours per week.

**A. Personal care (e.g bathing, dressing)**	1. ☐ Yes 2. ☐ No	.... Hours per weeks
**B. Housework / laundry**	1. ☐ Yes 2. ☐ No	.... Hours per weeks
**C. Providing transport / taking you out**	1. ☐ Yes 2. ☐ No	.... Hours per weeks
**D. Preparing meals**	1. ☐ Yes 2. ☐ No	.... Hours per weeks
**E. Gardening**	1. ☐ Yes 2. ☐ No	.... Hours per weeks
**F. Shopping**	1. ☐ Yes 2. ☐ No	.... Hours per weeks
**G. Looking after pets**	1. ☐ Yes 2. ☐ No	.... Hours per weeks
**H. DIY / home improvements**	1. ☐ Yes 2. ☐ No	.... Hours per weeks
**I. Other (please describe)**		.... Hours per weeks

In the last 6 months, have friends or relatives stayed off work to help you? Please include any time they took off to look after you, take you to heath care appointments or visit you in hospital in hours or days

1. ☐ Yes .... hours .... days

2. ☐ No

□□□□□: □□

EQ-5D 5L

Under each heading, please tick the ONE box that best describes your health **TODAY**

MOBILITY
I have no problems in walking about ☐
I have slight problems in walking about ☐
I have moderate problems in walking about ☐
I have severe problems in walking about ☐
I am unable to walk about ☐

SELF-CARE
I have no problems washing or dressing myself ☐
I have slight problems washing or dressing myself ☐
I have moderate problems washing or dressing myself ☐
I have severe problems washing or dressing myself ☐
I am unable to wash or dress myself ☐

USUAL ACTIVITIES *(e.g. work, study, housework, family or leisure activities)*
I have no problems doing my usual activities ☐
I have slight problems doing my usual activities ☐
I have moderate problems doing my usual activities ☐
I have severe problems doing my usual activities ☐
I am unable to do my usual activities ☐

PAIN / DISCOMFORT
I have no pain or discomfort ☐
I have slight pain or discomfort ☐
I have moderate pain or discomfort ☐
I have severe pain or discomfort ☐
I have extreme pain or discomfort ☐

ANXIETY / DEPRESSION
I am not anxious or depressed ☐
I am slightly anxious or depressed ☐
I am moderately anxious or depressed ☐
I am severely anxious or depressed ☐
I am extremely anxious or depressed ☐

□□□□□: □□

THE FUNCTIONAL MOBILITY SCALE

Please tick one box for each question:

✓	**1. How do you move around for short distances in the house? (5 metres/yards)**
	I use wheelchair
	I use a walker or frame and I need some help from another person
	I use crutches without help from another person
	I use sticks (one or 2), without help from another person
	I am independent on level surfaces, I don't use walking aids, I need to hold onto a rail when I use stairs
	I am independent on all surfaces, I don't need any walking aids or help from another person, I am able to walking in a crowded area without walking aids or help

✓	**2. How do you move around for medium distances (e.g. at school or at work)? (50m)**
	I use wheelchair
	I use a walker or frame and I need some help from another person
	I use crutches without help from another person
	I use sticks (one or 2), without help from another person
	I am independent on level surfaces, I don't use walking aids, I need to hold onto a rail when I use stairs
	I am independent on all surfaces, I don't need any walking aids or help from another person, I am able to walking in a crowded area without walking aids or help

✓	**3. How do you move around for long distance in a community setting (e.g. shopping centre)? (500m)**
	I use wheelchair
	I use a walker or frame and I need some help from another person
	I use crutches without help from another person
	I use sticks (one or 2), without help from another person
	I am independent on level surfaces, I don't use walking aids, I need to hold onto a rail when I use stairs
	I am independent on all surfaces, I don't need any walking aids or help from another person, I am able to walking in a crowded area without walking aids or help

✓	**4. Have you fallen over because of your movement problem?**
	**Yes**
	**No**

Please describe the worst injury you have had from a fall

____
